# Synthesis and reactivity of BINEPINE-based chiral Fe(II) PNP pincer complexes

**DOI:** 10.1007/s00706-016-1706-x

**Published:** 2016-03-21

**Authors:** Christian Schröder-Holzhacker, Nikolaus Gorgas, Berthold Stöger, Karl Kirchner

**Affiliations:** Institute of Applied Synthetic Chemistry, Vienna University of Technology, Getreidemarkt 9/163-OC, 1060 Vienna, Austria; Institute of Chemical Technologies and Analytics, Vienna University of Technology, Getreidemarkt 9, 1060 Vienna, Austria

**Keywords:** Iron, Pincer ligands, Chiral phosphines, Carbon monoxide

## Abstract

**Abstract:**

A new asymmetric chiral PNP ligand based on the 2,6-diaminopyridine scaffold featuring a *R*-BINEPINE moiety was prepared. Treatment of anhydrous FeX_2_ (X = Cl, Br) with 1 equiv of PNP-*i*Pr,BIN at room temperature afforded the coordinatively unsaturated paramagnetic complexes [Fe(PNP-*i*Pr,BIN)X_2_]. The structure of [Fe(PNP-*i*Pr,BIN)Cl_2_] is described. Both complexes react readily with the strong π-acceptor ligand CO in solution to afford selectively the diamagnetic complexes *trans*-[Fe(PNP-*i*Pr,BIN)(CO)X_2_] in quantitative yield. Due the lability of the CO ligand, these complexes are only stable under a CO atmosphere and isolation in pure form was not possible. The preparation of the carbonyl hydride complex [Fe(PNP-*i*Pr,BIN)(H)(CO)Br] was achieved albeit in low yields via a one pot procedure by treatment of [Fe(PNP-*i*Pr,BINEP)Br_2_] with CO and subsequent reaction with Na[HBEt_3_]. This complex was obtained as an inseparable mixture of two diastereomers in a ca. 1:1 ratio and was tested as catalyst for the hydrogenation of ketones. The catalyst showed acceptable activity under mild conditions (5 bar H_2_, room temperature) with yields up to >99 % within 18 h.

**Graphical abstract:**

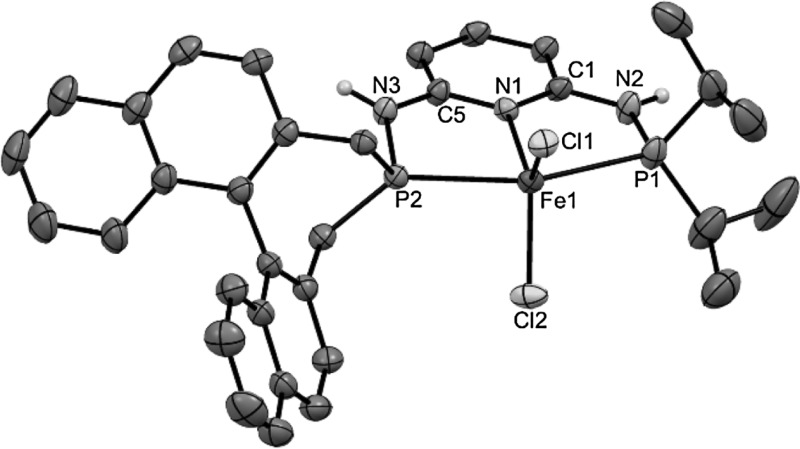

**Electronic supplementary material:**

The online version of this article (doi:10.1007/s00706-016-1706-x) contains supplementary material, which is available to authorized users.

## Introduction

In view of concerns regarding economy, environment, and sustainable energy, there is a constant need for the discovery of new catalytic reactions. A process we are interested in is the catalytic hydrogenation of polar multiple bonds via molecular hydrogen. This plays a significant role in modern synthetic organic chemistry and is excellently performed by many transition metal hydride complexes containing noble metals such as ruthenium, rhodium, or iridium [1–5]. The limited availability of precious metals, their high price, and their toxicity lowers the attractivity of these metals in the future and more economical and environmentally friendly alternatives have to be found which are in line with green chemistry guidelines. In this respect, the preparation of well-defined iron-based hydride catalysts of comparable or even higher activity is desirable [6–12]. Iron is the most abundant transition metal in the earth crust, and ubiquitously available. Accordingly, it is not surprising that the field of iron catalyzed hydrogenations of polar multiple bonds is rapidly evolving as shown by several recent examples [13–27].

We are currently focusing on the synthesis and reactivity of iron complexes containing PNP pincer ligands based on the 2,6-diaminopyridine scaffold [28–38]. In these ligands the aromatic pyridine ring and the phosphine moieties are connected via NH, N-alkyl, or N-aryl linkers. Complexes of the type [Fe(PNP-*i*Pr)(H)(CO)(L)]^n^ with labile ligands (L = Br^−^, CH_3_CN, BH_4_^−^) and NH spacers were found to be efficient catalysts for the hydrogenation of both ketones and aldehydes to alcohols under mild conditions (Scheme 1) [35].

**Figure:**
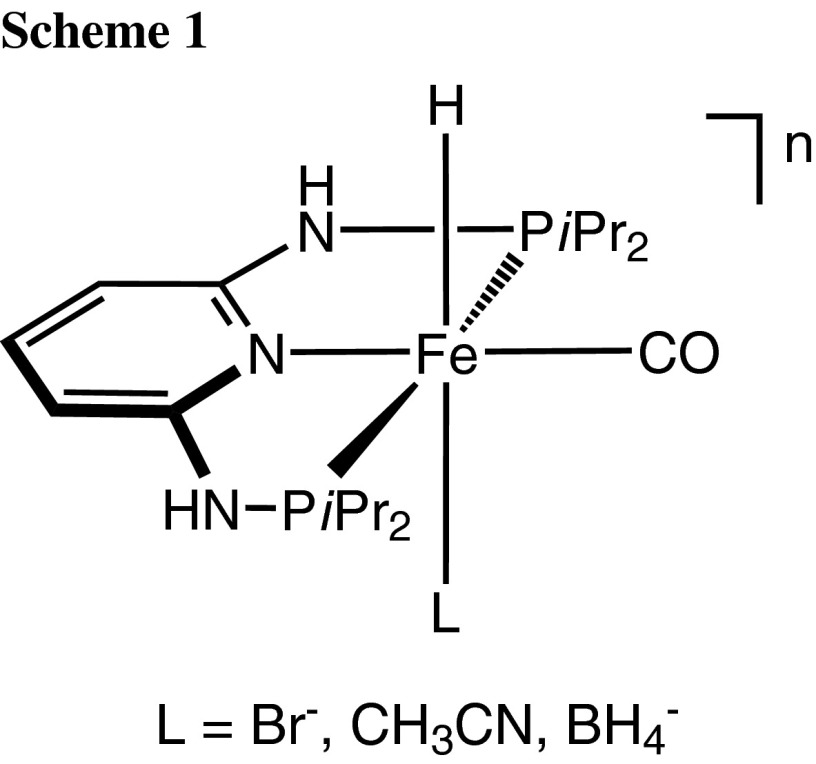

Herein we report on the synthesis, characterization, and preliminary catalytic activity of asymmetric chiral iron pincer complexes where P*i*Pr_2_ and BINEPINE moieties are connected to the pyridine ring of the PNP ligand via NH spacers. The *i*Pr substituents were chosen to prevent the coordination of two PNP ligands as found recently for sterically little demanding PNP systems [34, 36].

## Results and discussion

Treatment of the mono-phosphinated ligand PN^NH2^-*i*Pr (**1**) with 1 equiv of BIN-PCl (**2**) (in the *R*-form) in the presence of NEt_3_ afforded the new asymmetric chiral PNP ligand PNP-*i*Pr,BIN (**3**) in 64 % isolated yield (Scheme 2). The optically pure ligand was isolated as air stable solid and was characterized by elemental analysis, ^1^H, ^13^C{^1^H}, and ^31^P{^1^H} NMR spectroscopy.

**Figure:**
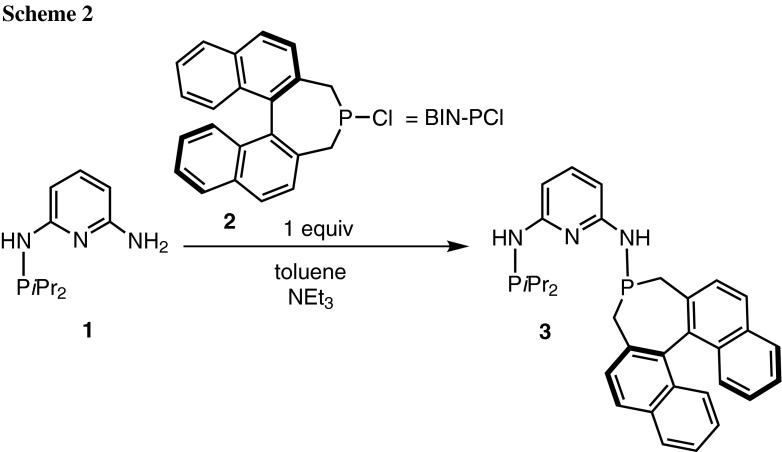

**Fig. 1:**
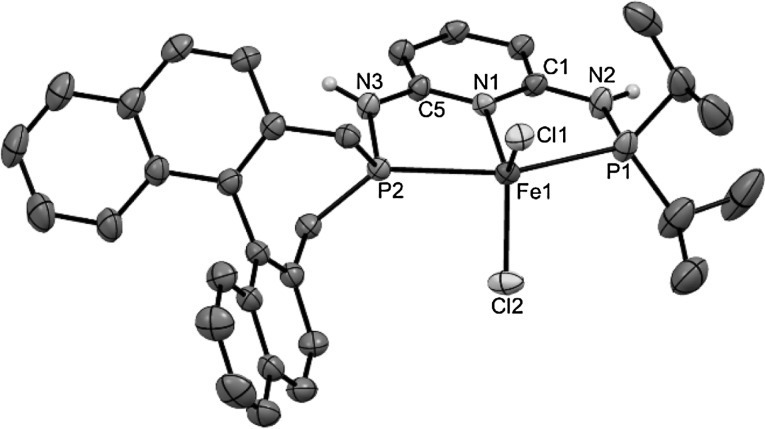
Structural view of [Fe(PNP-*i*Pr,BIN)Cl_2_] (**4a**) showing 50 % thermal ellipsoids (most H atoms and solvent molecules omitted for clarity). Selected bond (Å) and bond angles (°): Fe1–Cl1 2.3057(8), Fe1–Cl2 2.3366(9), Fe1–P1 2.4853(9), Fe1–P2 2.5150(9), Fe1–N1 2.141(2), Cl1–Fe1–Cl2 130.99(3), Cl1–Fe1–P1 97.69(3), Cl2–Fe1–P2 92.32(3), P1–Fe1–P2 159.16(3), Cl1–Fe1–P1–N2 130.5(1), Cl2–Fe1–P1–N2 97.0(1)

Treatment of anhydrous FeCl_2_ with 1 equiv of the PNP ligand PNP-*i*Pr,BIN (**3**) in THF at room temperature afforded the coordinatively unsaturated complex [Fe(PNP-*i*Pr,BIN)Cl_2_] (**4a**) in 79 % isolated yields (Scheme 3). The analogous bromide complex [Fe(PNP-*i*Pr,BIN)Br_2_] (**4b**) was obtained in similar fashion by straightforward complexation of the respective free PNP ligand with anhydrous ferrous dibromide (84 % yield). All complexes are thermally robust pale yellow solids that are air sensitive in the solid state and particularly in solution. They display large paramagnetic shifted ^1^H and ^13^C{^1^H} NMR solution spectra with broad and featureless signals which, due to the complexity of the PNP ligands, were not assignable and thus not informative. These complexes were characterized by elemental analysis. In addition the molecular structure of **4d** was determined by X-ray crystallography. In this case, the racemic BIN-PCl (**2**) was used, since all attempts to grow crystals suitable for X-ray diffraction studies failed for the chiral compounds. A structural view of **4a** is depicted in Fig. 1 with selected bond distances and angles given in the caption. The coordination geometry of the iron center is distorted square pyramidal. Bond distances and angles are in good accord with the solid state structures of similar complexes of the type [Fe(κ^3^*P,N,P*-PNP)Cl_2_] [29]. Particularly characteristic for all these complexes are the comparatively long Fe–N and Fe–P bonds which clearly indicate that they are in high-spin state; low-spin Fe–PNP complexes have Fe–N and Fe–P bonds by ca. 0.2 Å shorter.

**Figure:**
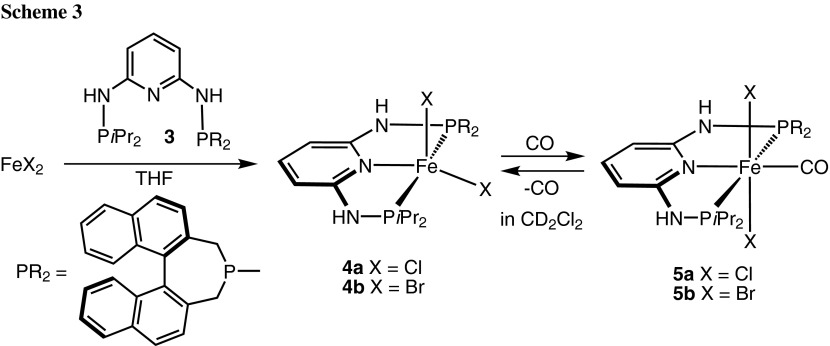

In order to obtain iron-based hydrogenation catalysts it appears to be important to have diamagnetic complexes. Accordingly, virtually all iron complexes that are active catalysts feature the strong field CO and hydride ligands, which seem to maintain a low spin configuration throughout the catalytic cycle. Complexes **4a** and **4b** react readily with the strong π-acceptor ligand CO in solution to afford selectively the diamagnetic, octahedral complexes *trans*-[Fe(PNP-*i*Pr,BIN)(CO)Cl_2_] (**5a**) and *trans*-[Fe(PNP-*i*Pr,BIN)(CO)Br_2_] (**5b**), respectively, in quantitative yield (Scheme 3). However, due the lability of the CO ligand, these complexes are stable only under a CO atmosphere and isolation in pure form was not possible. These compounds slowly release CO thereby reforming the starting materials **4a** and **4b**. Accordingly, complexes **5a** and **5b** were fully characterized by ^1^H, ^13^C{^1^H}, and ^31^P{^1^H} NMR spectroscopy. In the ^13^C{^1^H} NMR spectrum the CO ligands of **5a** and **5b** exhibit a single low-intensity triplet resonance at 220.7 (t, ^*2*^*J*_*CP*_ = 22.2 Hz) and 223.1 ppm (t, ^*2*^*J*_*CP*_ = 21.9 Hz), respectively. The ^31^P{^1^H} NMR spectra give rise to two doublets centered at 143.6/125.3 and 143.9/125.1 ppm, respectively, with large *J*_*PP*_ coupling constants of 190 and 177 Hz which are consistent with a *trans*-P,P configuration.

**Fig. 2:**
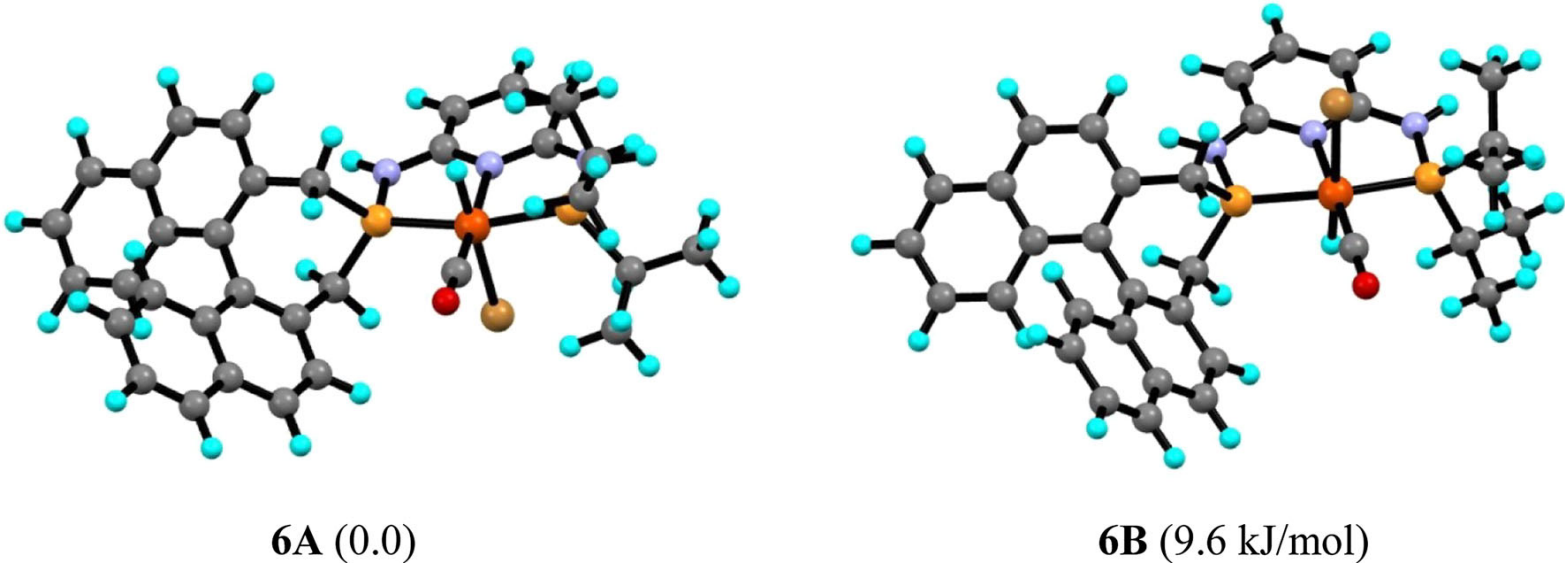
DFT/B3LYP optimized structures and free energy difference of the two diasteromers of [Fe(PNP-*i*Pr,BIN)(H)(CO)Br] (**6**)

**Fig. 3:**
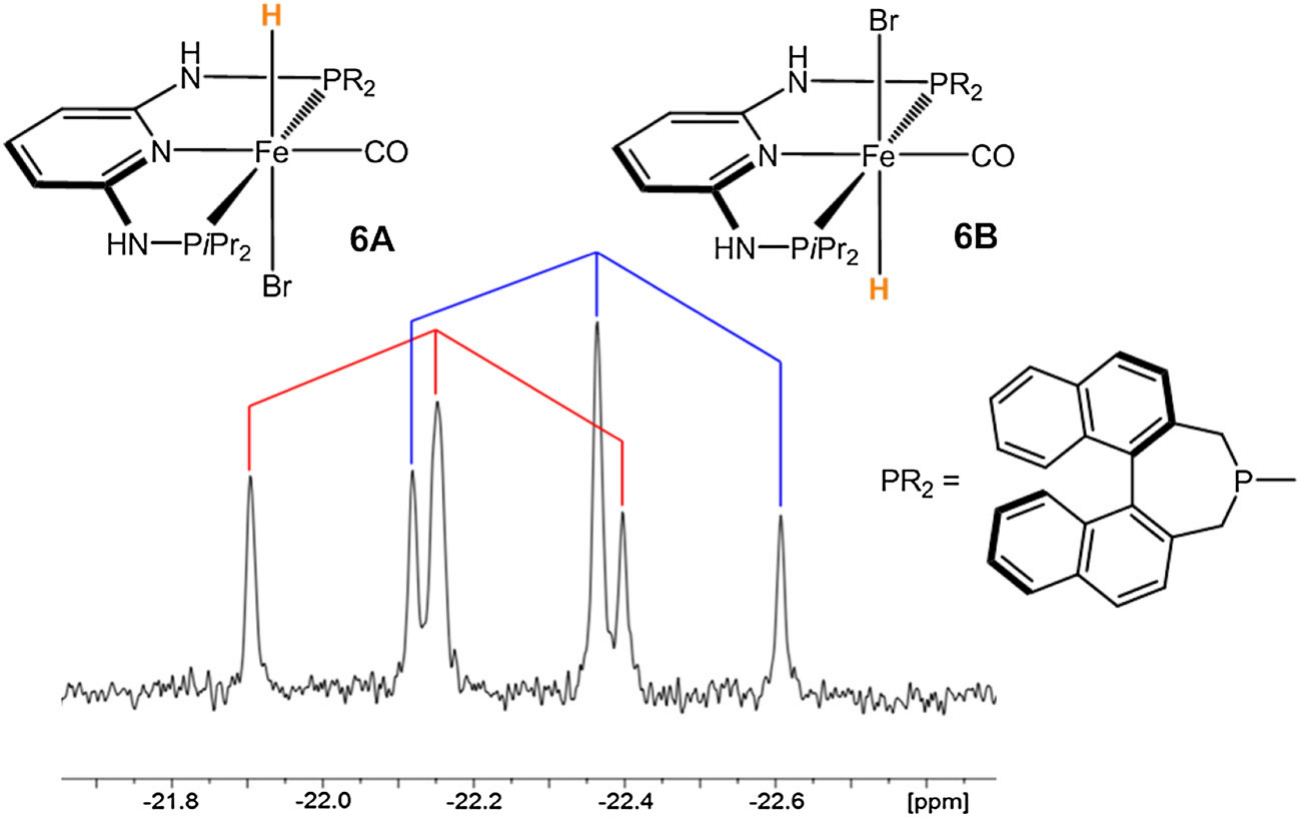
Hydride region of the ^1^H NMR spectrum of **6A** (*left*) and **6B** (*right*) in CD_3_OD

The preparation of carbonyl hydride complexes was attempted via a one pot procedure by treatment of [Fe(PNP-*i*Pr,BINEP)Br_2_] (**4b**) with CO and subsequent reaction with 1.1 equivs of Na[HBEt_3_] (Scheme 4). Complex [Fe(PNP-*i*Pr,BIN)(H)(CO)Br] (**6**) was obtained as an inseparable mixture of two diastereomers in a ca. 1:1 ratio. Together with **6**, a considerable amount of free PNP-*i*Pr,BIN ligand was detected in the ^31^P{^1^H} NMR spectrum, and the yield was low (ca 50 %). In good agreement with the experimental findings, also DFT/B3LYP calculations showed that the energy difference between the two isomers (denoted as **6A** and **6B**) is merely 10.5 kJ/mol (Fig. 2). In both isomers, the hydride ligands are located *trans* to the bromide ligand. Complex **6** turned out to be unstable in solution and slow decomposition took place. Accordingly, complete purification and characterization was unsuccessful. The products were identified by ^1^H NMR where the hydride signals of the two isomers **6A** and **6B** gave rise to triplet resonances at −22.15 (^*2*^*J*_*PH*_ = 61.6 Hz) and −22.36 ppm (^*2*^*J*_*PH*_ = 61.1 Hz), respectively (Fig. 3). The assignments are based on DFT calculations.

**Figure:**
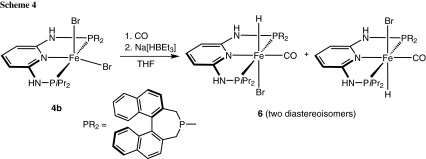

Despite these facts, complex **6** (as mixture of two diastereomers), prepared in situ, was tested as catalyst for the hydrogenation of some ketones. Since the catalyst could not be isolated in pure form, only preliminary tests were performed with five substrates. These results are depicted in Table 1. The catalyst showed acceptable activity under mild conditions (5 bar H_2_, room temperature) with yields up to >99 % within 18 h indicating that in further experiments the catalyst loadings and possibly reaction times can be reduced significantly.

**Table 1:**
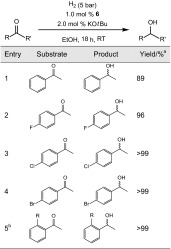
Hydrogenation of ketones catalyzed by [Fe(PNP-*i*Pr,BIN)(H)(CO)Br] (**6**)

^a^Yields were determined by ^1^H NMR

^b^ *R* = naphtyl

In conclusion, we describe here the synthesis of a new asymmetric chiral PNP ligand based on the 2,6-diaminopyridine scaffold featuring an *R*-BINEPINE moiety. This ligand reacts with anhydrous FeX_2_ (X = Cl, Br) to afford the coordinatively unsaturated paramagnetic complexes [Fe(PNP-*i*Pr,BIN)X_2_]. Both complexes react readily with the strong π-acceptor ligand CO in solution to afford selectively and quantitatively *trans*-[Fe(PNP-*i*Pr,BIN)(CO)X_2_]. Due the lability of the CO ligand, these complexes are only stable under a CO atmosphere. The preparation of the carbonyl hydride complex [Fe(PNP-*i*Pr,BIN)(H)(CO)Br] was achieved albeit in low yields via a one pot synthesis. This complex was obtained as an inseparable mixture of two diastereomers and was successfully tested as catalyst for the hydrogenation of ketones.

## Experimental

All manipulations were performed under an inert atmosphere of argon by using Schlenk techniques or in an MBraun inert-gas glovebox. The solvents were purified according to standard procedures [39]. The deuterated solvents were purchased from Aldrich and dried over 4 Å molecular sieves. The ligands *N*^2^-(diisopropylphosphanyl)pyridine-2,6-diamine (PN^NH2^-*i*Pr) (**1**) and (1*R*)-4-chloro-4,5-dihydro-3*H*-dinaphto[2,1-*c*:1′,2′-*e*]phosphepine (BIN-PCl) (**2**) were prepared according to the literature [37, 40]. ^1^H, ^13^C{^1^H}, and ^31^P{^1^H} NMR spectra were recorded on Bruker AVANCE-250, AVANCE-300 DPX, and AVANCE-400 spectrometers. ^1^H and ^13^C{^1^H} NMR spectra were referenced internally to residual protio-solvent, and solvent resonances, respectively, and are reported relative to tetramethylsilane (*δ* = 0 ppm). ^31^P{^1^H} NMR spectra were referenced externally to H_3_PO_4_ (85 %) (*δ* = 0 ppm).

### *(1R)*-*N*^*2*^-*(3,5*-*Dihydro*-*4H*-*dinaphtho[2,1*-*c:1′,2′*-*e]phosphepin*-*4*-*yl)*-*N*^*6*^-*(diisopropylphosphanyl)pyridine*-*2,6*-*diamine (PNP*-*iPr,BIN)* (**3**, C_33_H_35_N_3_P_2_)

**1** (1.00 eq, 6.55 mmol, 1.47 g) was dissolved in 100 cm^3^ toluene and 1.4 cm^3^ Et_3_N (1.50 eq, 9.83 mmol) was added. After cooling to 0 °C, 2.50 g **2** (1.10 eq, 7.21 mmol) in 50 cm^3^ toluene was added and the reaction was stirred at 80 °C for 12 h. The suspension was filtered over a small pad of Celite^®^ and the solvent was removed under reduced pressure. The crude product was purified via flash chromatography using silica gel (conditioned with 5 vol % NEt_3_) and 1:1 MC/EE as eluent. Yield: 2.25 g (64 %). ^1^H NMR (CDCl_3_, 20 °C): *δ* = 7.89–7.79 (m, 4H, naph), 7.47 (d, ^*3*^*J*_*HH*_ = 8.3 Hz, 1H, naph), 7.38–7.32 (m, 2H, naph), 7.31 (d, ^*3*^*J*_*HH*_ = 8.3 Hz, 1H, naph), 7.23 (m, 5H, py^4^, naph), 6.43 (dd, ^*3*^*J*_*HH*_ = 8.0 Hz, ^*4*^*J*_*PH*_ = 1.9 Hz, 1H, py^5^), 6.22 (d, ^*3*^*J*_*HH*_ = 8.4 Hz, 1H, py^3^), 4.31 (d, ^*2*^*J*_*PH*_ = 10.9 Hz, 1H, N*H*^BIN^), 4.08 (d, ^*2*^*J*_*PH*_ = 10.7 Hz, 1H, N*H*^*i*Pr^), 3.03 (dd, ^*2*^*J*_*HH*_ = 17.0 Hz, ^*2*^*J*_*PH*_ = 11.9 Hz, 1H, C*H*_*2*_), 2.70 (dd, ^*2*^*J*_*HH*_ = 14.3 Hz, ^*2*^*J*_*PH*_ = 2.8 Hz, 1H, C*H*_*2*_), 2.50 (dd, ^*2*^*J*_*HH*_ = 18.2 Hz, ^*2*^*J*_*PH*_ = 14.3 Hz, 1H, C*H*_*2*_), 2.29 (d, ^*2*^*J*_*HH*_ = 11.9 Hz, 1H, C*H*_*2*_), 1.68 (m, 2H, C*H*(CH_3_)_2_), 1.04–0.95 (m, 12H, CH(C*H*_*3*_)_2_) ppm; ^13^C{^1^H} NMR (CDCl_3_, 20 °C): *δ* = 159.92 (d, ^*2*^*J*_*CP*_ = 20.0 Hz, py^6^), 157.01 (d, ^*2*^*J*_*CP*_ = 17.4, py^2^), 139.25 (s, py^4^), 133.79 (d, *J*_*CP*_ = 4.1 Hz, naph), 133.43 (s, naph), 133.08 (s, naph), 132.82 (d, *J*_*CP*_ = 1.5 Hz, naph), 132.72 (s, naph), 132.38 (s, naph), 132.28 (d, *J*_*CP*_ = 1.8 Hz, naph), 132.15 (s, naph), 128.24 (s, naph), 127.58 (s, naph), 127.23 (d, *J*_*CP*_ = 2.1 Hz, naph), 126.69 (d, *J*_*CP*_ = 10.8 Hz, naph), 126.06 (d, *J*_*CP*_ = 11.5 Hz, naph), 125.09 (d, *J*_*CP*_ = 11.9 Hz, naph), 98.90 (d, ^*3*^*J*_*CP*_ = 18.4 Hz, py^5^), 98.73 (d, ^*3*^*J*_*CP*_ = 15.7, py^3^), 36.04 (d, ^*1*^*J*_*CP*_ = 15.0 Hz, *C*H_2_), 34.90 (d, ^*1*^*J*_*CP*_ = 24.2 Hz, *C*H_2_), 26.43 (d, ^*1*^*J*_*CP*_ = 11.5 Hz, *C*H(CH_3_)_2_), 16.34 (d, ^*1*^*J*_*CP*_ = 11.0 Hz, *C*H(CH_3_)_2_), 18.72 (d, ^*2*^*J*_*CP*_ = 19.8 Hz, CH(*C*H_3_)_2_), 17.22 (d, ^*2*^*J*_*CP*_ = 10.6 Hz, CH(*C*H_3_)_2_), 17.14 (d, ^*2*^*J*_*CP*_ = 10.4 Hz, CH(*C*H_3_)_2_) ppm; ^31^P{^1^H} NMR (CDCl_3_, 20 °C): *δ* = 48.6 (s, *i*Pr), 48.1 (s, BIN) ppm.

### *[(Dichloro)(1R)*-*N*^*2*^-*(3,5*-*dihydro*-*4H*-*dinaphtho[2,1*-*c:1′,2′*-*e]phosphepin*-*4*-*yl)*-*N*^*6*^-*(diisopropylphosphanyl)pyridine*-*2,6*-*diamine)iron(II)] ([Fe(PNP*-*iPr,BIN)Cl*_*2*_*])* (**4a**, C_33_H_35_Cl_2_FeN_3_P_2_)

A suspension of 71 mg anhydrous FeCl_2_ (0.56 mmol) and 300 mg **3** (0.56 mmol) was stirred in 15 cm^3^ of THF at room temperature for 12 h. The solvent was then removed under vacuum and the remaining solid dissolved in 15 cm^3^ of CH_2_Cl_2_. Insoluble materials were removed by filtration. The volume of the solution was reduced to 0.5 cm^3^ and the product was precipitated by addition of 40 cm^3^ of *n*-pentane. After filtration the yellow product was washed with twice with 15 cm^3^ of *n*-pentane and dried under vacuum. Yield: 324 mg (87 %) yellow solid.

### *[(Dibromo)(1R)*-*N*^*2*^-*(3,5*-*dihydro*-*4H*-*dinaphtho[2,1*-*c:1′,2′*-*e]phosphepin*-*4*-*yl)*-*N*^*6*^-*(diisopropylphosphanyl)pyridine*-*2,6*-*diamine)iron(II)] ([Fe(PNP*-*iPr,BIN)Br*_*2*_*])* (**4b**, C_33_H_35_Br_2_FeN_3_P_2_)

This complex was prepared analogously to **4a** with 121 mg anhydrous FeBr_2_ (0.56 mmol) and 300 mg **3** (0.56 mmol) as starting materials. Yield: 352 mg (84 %), yellow solid.

### *Reaction of [Fe(PNP*-*iPr,BIN)Cl*_*2*_*] (****4a****) with CO in CD*_*2*_*Cl*_*2*_*. Formation of trans*-*[(dichloro)(carbonyl)(1R)*-*N*^*2*^-*(3,5*-*dihydro*-*4H*-*dinaphtho[2,1*-*c:1′,2′*-*e]phosphepin*-*4*-*yl)*-*N*^*6*^-*(diisopropylphosphanyl)pyridine*-*2,6*-*diamine)iron(II)] (trans*-*[Fe(PNP*-*iPr,BIN)(CO)Cl*_*2*_*])* (**5a**, C_34_H_35_Cl_2_FeN_3_OP_2_)

CO was bubbled through a solution of 30 mg **4a** (45.3 µmol) in 0.6 cm^3^ of CD_2_Cl_2_ for 2 min, whereupon the colour changed to dark violet. ^1^H NMR (CD_2_Cl_2_, 20 °C): *δ* = 8.13–8.00 (m, 4H, naph), 7.79 (d, ^*3*^*J*_*HH*_ = 7.9 Hz, 1H, naph), 7.66 (d, ^*3*^*J*_*HH*_ = 7.5 Hz, 1H, naph), 7.41 (d, ^*3*^*J*_*HH*_ = 8.4 Hz, 1H, naph), 7.34–7.15 (m, 6H, naph, py^4^), 6.62 (bs, 1H, py^5^), 6.41 (bs, 1H, N*H*^iPr^), 6.03 (bs, 1H, py^3^), 5.56 (bs, 1H, N*H*^BIN^), 4.35 (dd, ^*2*^*J*_*HH*_ = 12.3 Hz, ^*2*^*J*_*PH*_ = 4.1 Hz, 1H, C*H*_*2*_), 3.76 (dd, ^*2*^*J*_*HH*_ = 15.1 Hz, ^*2*^*J*_*PH*_ = 9.1 Hz, 1H, C*H*_*2*_), 3.20 (d, ^*2*^*J*_*HH*_ = 15.0 Hz, 1H, C*H*_*2*_), 2.99 (m, 2H, C*H*(CH_3_)_2_), 2.71 (dd, ^*2*^*J*_*HH*_ = 17.0 Hz, ^*2*^*J*_*PH*_ = 13.0 Hz, 1H, C*H*_*2*_), 1.62–1.37 (m, 12H, CH(C*H*_*3*_)_2_) ppm; ^13^C{^1^H} NMR (CD_2_Cl_2_, 20 °C): *δ* = 220.73 (t, ^*2*^*J*_*CP*_ = 22.2 Hz, *C*O), 161.90 (dd, ^*2*^*J*_*CP*_ = 13.2 Hz, ^*3*^*J*_*CP*_ = 5.6 Hz, py^6^), 160.63 (dd, ^*2*^*J*_*CP*_ = 13.6 Hz, ^*3*^*J*_*CP*_ = 5.1 Hz, py^2^), 140.16 (s, py^4^), 134.67 (d, *J*_*CP*_ = 1.8 Hz, naph), 134.16 (d, *J*_*CP*_ = 4.9 Hz, naph), 133.21 (d, *J*_*CP*_ = 2.6 Hz, naph), 132.91 (d, *J*_*CP*_ = 1.6 Hz, naph), 132.82 (s, naph), 132.49 (s, naph), 132.24 (d, *J*_*CP*_ = 2.5 Hz, naph), 131.56 (d, *J*_*CP*_ = 3.0 Hz, naph), 129.03 (s, naph), 128.81 (d, *J*_*CP*_ = 2.1 Hz, naph), 128.45 (d, *J*_*CP*_ = 3.6 Hz, naph), 128.37 (d, *J*_*CP*_ = 8.0 Hz, naph), 127.53 (s, naph), 126.96 (s, naph), 126.79 (s, naph), 126.40 (s, naph), 126.08 (s, naph), 125.65 (s, naph), 125.51 (s, naph), 99.88 (d, ^*3*^*J*_*CP*_ = 7.7 Hz, py^5^), 99.52 (d, ^*3*^*J*_*CP*_ = 7.2 Hz, py^3^), 33.05 (d, ^*1*^*J*_*CP*_ = 21.9 Hz, *C*H_2_), 29.10 (d, ^*1*^*J*_*CP*_ = 26.3 Hz, *C*H_2_), 26.23 (d, ^*1*^*J*_*CP*_ = 21.9 Hz, *C*H(CH_3_)_2_), 25.95 (d, ^*1*^*J*_*CP*_ = 22.2 Hz, *C*H(CH_3_)_2_), 18.88 (d, ^*2*^*J*_*CP*_ = 4.1 Hz, CH(*C*H_3_)_2_), 18.86 (d, ^*2*^*J*_*CP*_ = 4.0 Hz, CH(*C*H_3_)_2_), 17.95 (d, ^*2*^*J*_*CP*_ = 3.5 Hz, CH(*C*H_3_)_2_) ppm; ^31^P{^1^H} NMR (CD_2_Cl_2_, 20 °C): *δ* = 143.6 (d, ^*2*^*J*_*PP*_ = 189.7 Hz, BIN), 125.3 (d, ^*2*^*J*_*PP*_ = 189.7 Hz, *i*Pr) ppm.

### *Reaction of [Fe(PNP*-*iPr,BIN)Br*_*2*_*] (****4b****) with CO in CD*_*2*_*Cl*_*2*_*. Formation of trans*-*[(dibromo)(carbonyl)(1R)*-*N*^*2*^-*(3,5*-*dihydro*-*4H*-*dinaphtho[2,1*-*c:1′,2′*-*e]phosphepin*-*4*-*yl)*-*N*^*6*^-*(diisopropylphosphanyl)pyridine*-*2,6*-*diamine)iron(II)] (trans*-*[Fe(PNP*-*iPr,BIN)(CO)Br*_*2*_*])* (**5b**, C_34_H_35_Br_2_FeN_3_OP_2_)

CO was bubbled through a solution of 30 mg **4b** (39.9 µmol) in 0.6 cm^3^ of CD_2_Cl_2_ for 2 min, whereupon the colour changed to dark violet. ^1^H NMR (CD_2_Cl_2_, 20 °C): *δ* = 8.01–7.90 (m, 4H, naph), 7.80 (d, ^*3*^*J*_*HH*_ = 7.9 Hz, 1H, naph), 7.54 (d, ^*3*^*J*_*HH*_ = 7.5 Hz, 1H, naph), 7.41–7.36 (m, 3H, naph), 7.30 (d, ^*3*^*J*_*HH*_ = 8.4 Hz, 1H, naph), 7.25–7.03 (m, 3H, naph, py^4^), 6.50 (bs, 1H, py^5^), 6.32 (d, ^*2*^*J*_*PH*_ = 5.9 Hz, 1H, N*H*^iPr^), 5.83 (bs, 1H, py^3^), 5.50 (d, ^*2*^*J*_*PH*_ = 6.0 Hz, 1H, N*H*^BIN^), 4.67 (dd, ^*2*^*J*_*HH*_ = 12.5 Hz, ^*2*^*J*_*PH*_ = 3.9 Hz, 1H, C*H*_*2*_), 3.94 (dd, ^*2*^*J*_*HH*_ = 15.1 Hz, ^*2*^*J*_*PH*_ = 8.8 Hz, 1H, C*H*_*2*_), 3.10 (m, 3H, C*H*_*2*_, C*H*(CH_3_)_2_), 2.73 (dd, ^*2*^*J*_*HH*_ = 17.3 Hz, ^*2*^*J*_*PH*_ = 13.3 Hz, 1H, C*H*_*2*_), 1.49 (dd, ^*3*^*J*_*HH*_ = 5.8 Hz, ^*2*^*J*_*PH*_ = 12.1 Hz, 3H, CH(C*H*_*3*_)_2_), 1.45 (dd, ^*3*^*J*_*HH*_ = 6.8 Hz, ^*2*^*J*_*PH*_ = 15.3 Hz, 3H, CH(C*H*_*3*_)_2_), 1.41 (dd, ^*3*^*J*_*HH*_ = 7.2 Hz, ^*2*^*J*_*PH*_ = 16.9 Hz, 3H, CH(C*H*_*3*_)_2_), 1.32 (dd, ^*3*^*J*_*HH*_ = 7.2 Hz, ^*2*^*J*_*PH*_ = 16.6 Hz, 3H, CH(C*H*_*3*_)_2_) ppm; ^13^C{^1^H} NMR (CD_2_Cl_2_, 20 °C): *δ* = 223.05 (t, ^*2*^*J*_*CP*_ = 21.9 Hz, *C*O), 161.97 (dd, ^*2*^*J*_*CP*_ = 12.6 Hz, ^*3*^*J*_*CP*_ = 5.3 Hz, py^6^), 160.60 (dd, ^*2*^*J*_*CP*_ = 13.3 Hz, ^*3*^*J*_*CP*_ = 5.1 Hz, py^2^), 140.11 (s, py^4^), 134.65 (s, naph), 134.12 (d, *J*_*CP*_ = 5.0 Hz, naph), 133.67 (d, *J*_*CP*_ = 10.9 Hz, naph), 133.18 (d, *J*_*CP*_ = 2.6 Hz, naph), 132.92 (d, *J*_*CP*_ = 1.3 Hz, naph), 132.44 (s, naph), 132.18 (d, *J*_*CP*_ = 2.4 Hz, naph), 131.61 (d, *J*_*CP*_ = 2.9 Hz, naph), 129.08 (s, naph), 128.80 (d, *J*_*CP*_ = 2.2 Hz, naph), 128.39 (d, *J*_*CP*_ = 6.2 Hz, naph), 128.22 (d, *J*_*CP*_ = 3.5 Hz, naph), 127.63 (d, *J*_*CP*_ = 1.6 Hz, naph), 126.92 (d, *J*_*CP*_ = 13.8 Hz, naph), 126.41 (s, naph), 126.10 (s, naph), 125.61 (d, *J*_*CP*_ = 13.5 Hz, naph), 100.17 (d, ^*3*^*J*_*CP*_ = 7.0 Hz, py^5^), 99.67 (d, ^*3*^*J*_*CP*_ = 7.2 Hz, py^3^), 36.23 (d, ^*1*^*J*_*CP*_ = 23.3 Hz, *C*H_2_), 31.39 (d, ^*1*^*J*_*CP*_ = 27.9 Hz, *C*H_2_), 28.48 (d, ^*1*^*J*_*CP*_ = 22.6 Hz, *C*H(CH_3_)_2_), 28.01 (d, ^*1*^*J*_*CP*_ = 23.3 Hz, *C*H(CH_3_)_2_), 18.97 (d, ^*2*^*J*_*CP*_ = 4.5 Hz, CH(*C*H_3_)_2_), 18.52 (m, CH(*C*H_3_)_2_) ppm; ^31^P{^1^H} NMR (CD_2_Cl_2_, 20 °C): *δ* = 143.9 (d, ^*2*^*J*_*PP*_ = 176.6 Hz, BIN), 125.1 (d, ^*2*^*J*_*PP*_ = 176.5 Hz, *i*Pr) ppm.

### *Reaction of [Fe(PNP*-*iPr,BIN)Br*_*2*_*] (****4b****) with CO and Na[HBEt*_*3*_*]. Formation of [(bromo)(hydrido)(carbonyl)(1R)*-*N*^*2*^-*(3,5*-*dihydro*-*4H*-*dinaphtho[2,1*-*c:1′,2′*-*e]phosphepin*-*4*-*yl)*-*N*^*6*^-*(diisopropylphosphanyl)pyridine*-*2,6*-*diamine)iron(II)] ([Fe(PNP*-*iPr,BIN)(H)(CO)Br])* (**6**, C_34_H_36_BrFeN_3_OP_2_)

A solution of 300 mg **4b** (0.40 mmol) in 15 cm^3^ THF was purged with CO for 3 min, whereupon the colour changed to deep blue. The reaction mixture was then cooled to 0 °C and 0.44 cm^3^ Na[HBEt_3_] (0.44 mmol) was added slowly via syringe. The solvent was then removed under reduced pressure. The residue was redissolved in 15 cm^3^ of CH_2_Cl_2_, filtered and the volume of the solution was reduced to ca 0.5 cm^3^. The product was precipitated upon addition of 40 cm^3^ of *n*-pentane, collected on a glass frit, washed with 10 cm^3^*n*-pentane and dried under vacuum for 2 h. The product could not be isolated in pure form and was used for catalytic reactions as obtained.

### X-ray structure determination

X-ray diffraction data of **4a**·*x*THF·(2 − *x*)Et_2_O (CCDC number 1445976) were collected at *T* = 100 K in a dry stream of nitrogen on a Bruker Kappa APEX II diffractometer system using graphite-monochromatized Mo*K*α radiation (*λ* = 0.71073 Å) and fine sliced *φ*- and *ω***-**scans. Data were reduced to intensity values with SAINT and an absorption correction was applied with the multi-scan approach implemented in SADABS [41]. The structures were solved by charge flipping using SUPERFLIP [42] and refined against *F* with JANA2006 [43]. The electron density in distinct voids of the structure could be attributed to two solvent positions. On one was located an Et_2_O molecule and the other was substitutionally disordered by Et_2_O and THF molecules. Since no refinement with reasonable ADPs of the solvent molecules could be obtained, contributions of the solvent molecules to the diffraction data were removed using the SQUEEZE procedure of PLATON [44]. The non-hydrogen atoms were refined anisotropically. The H atoms connected to C atoms were placed in calculated positions and thereafter refined as riding on the parent atoms. H atoms connected to N located in difference Fourier maps. Since the point group 4/*m* of the crystal is a merohedry twinning via twofold rotation about [110] was included in the model. Such models did not result in improved residuals and twinning was ultimately dropped from the refinement. Molecular graphics were generated with the program MERCURY [45]. Crystal data are given in Table S1.

### Computational details

Calculations were performed using the Gaussian 09 software package [46], and the B3LYP functional [47–49] without symmetry constraints. This functional was shown to perform well in mechanistic studies of spin forbidden reactions in closely related Fe system. The optimized geometries were obtained with the Stuttgart/Dresden ECP (SDD) basis set [50–52] to describe the electrons of the iron atom. For all other atoms a standard 6-31G** basis set was employed [53–58]. Frequency calculations were performed to confirm the nature of the stationary points yielding no imaginary frequency for the minima.

## Electronic supplementary material

Below is the link to the electronic supplementary material.

Supplementary material 1 (DOCX 48 kb)

Supplementary material 2 (CIF 553 kb)
