# Supplementary material for: Synthesis and reactivity of BINEPINE-based chiral Fe(II) PNP pincer complexes
Source: Monatsh Chem. 2016 Mar 21;147:1023–30. doi: 10.1007/s00706-016-1706-x (PMC4869728; doi:10.1007/s00706-016-1706-x)
Supplement: Supplementary file 1 — Supplementary material 1 (DOCX 48 kb) [file 706_2016_1706_MOESM1_ESM.docx]

**Synthesis and reactivity of BINEPINE-based chiral Fe(II) PNP pincer complexes**

**Christian Schröder-Holzhacker ● Nikolaus Gorgas ● Berthold Stöger ● Karl Kirchner***

**Supporting Information**

**Table S1.** Details for the crystal structure determination of **4a**·*x*THF·(2-*x*)Et_2_O.

|  | **4a**·*x*THF·(2-*x*)Et_2_O |
| --- | --- |
| formula | C_33_H_35_Cl_2_FeN_3_P_2_ |
| fw | 662.4 |
| cryst.size, mm | 0.54 x 0.44 x 0.31 |
| color, shape | translucent colourless, irregular |
| crystal system | tetragonal |
| space group | *I*4_1_/*a* (no. 88) |
| *a*, Å | 24.1896(7) |
| *c*, Å | 28.4068(17) |
| *V*, Å^3^ | 16621.9(12) |
| *T*, K | 100 |
| Z,Z' | 16,1 |
| *ρ*_calc_, g cm^-3^ | 1.0587 |
| *µ*, mm^-1^ (MoKα) | 0.590 |
| *F*(000) | 5504 |
| absorption corrections, *T*_min_-*T*_max_ | multi-scan, 0.74–0.83 |
| *θ* range, deg | 1.11–30.06 |
| no. of rflns measd | 219225 |
| *R*_int_ | 0.0465 |
| no. of rflns unique | 12180 |
| no. of rflns *I*>3*σ*(*I*) | 8658 |
| no. of params / restraints | 370 / 0 |
| *R* (*I* > 3*σ*(*I*)) ^a^ | 0.0763 |
| *R* (all data) | 0.0980 |
| *wR* (*I* > 3*σ*(*I*)) | 0.0947 |
| *wR* (all data) | 0.0967 |
| GooF | 3.50 |
| Diff.Four.peaks  min/max, eÅ^-3^ | -1.10 / 1.72 |
| CCDC no. | 1445976 |

**Atomic Coordinates for all the optimized molecules (B3LYP)**

[Fe(PNP-*i*Pr**,**BIN)(H)(CO)Br] (**6A**).

26 4.516311000 4.325924000 0.108609000

15 4.931126000 5.857341000 -1.466859000

15 4.107368000 2.465459000 1.232760000

7 5.176325000 3.010942000 -1.340231000

7 5.568520000 4.830211000 -2.713708000

7 4.687137000 1.281033000 0.114778000

6 5.640981000 3.468066000 -2.525071000

6 6.153793000 2.610446000 -3.510027000

6 6.174540000 1.246776000 -3.243602000

6 5.690666000 0.757001000 -2.035835000

6 5.195073000 1.678827000 -1.103290000

6 3.547546000 6.751849000 -2.372677000

6 2.556213000 5.759344000 -2.998196000

6 2.819745000 7.741630000 -1.446666000

6 6.323559000 7.104234000 -1.273840000

6 6.604476000 7.955932000 -2.520512000

6 6.186598000 7.966958000 -0.008414000

6 2.357513000 1.912799000 1.594296000

6 4.957538000 1.985671000 2.825252000

6 2.294155000 0.578019000 2.289809000

6 1.947710000 -0.577556000 1.538391000

6 1.946769000 -1.826632000 2.110657000

6 2.320766000 -1.998792000 3.468048000

6 2.362751000 -3.286580000 4.068134000

6 2.761337000 -3.445182000 5.374913000

6 3.142550000 -2.314602000 6.136826000

6 3.104096000 -1.052366000 5.588174000

6 2.680549000 -0.846233000 4.243761000

6 2.637535000 0.455526000 3.637284000

6 4.142164000 2.407017000 4.022674000

6 4.511594000 3.584143000 4.726574000

6 3.769602000 4.031469000 5.792092000

6 2.596720000 3.344209000 6.198735000

6 1.796026000 3.816235000 7.274014000

6 0.638957000 3.165687000 7.634874000

6 0.230018000 2.011351000 6.925162000

6 0.987993000 1.522131000 5.884805000

6 2.200598000 2.158773000 5.493601000

6 3.012947000 1.684403000 4.408174000

1 6.522440000 3.013016000 -4.446680000

1 6.568868000 0.558382000 -3.985373000

1 5.695055000 -0.302597000 -1.807409000

1 4.026661000 7.318729000 -3.182199000

1 3.050151000 5.062921000 -3.681331000

1 2.053924000 5.171206000 -2.224958000

1 1.794864000 6.307812000 -3.564783000

1 2.007779000 8.232220000 -1.995200000

1 2.377169000 7.228268000 -0.587594000

1 3.479657000 8.525843000 -1.067875000

1 7.166602000 6.425416000 -1.100394000

1 5.811584000 8.688493000 -2.706663000

1 7.536297000 8.515406000 -2.381150000

1 6.727551000 7.355778000 -3.429727000

1 7.093460000 8.569868000 0.115369000

1 5.340943000 8.659882000 -0.057976000

1 6.084010000 7.339636000 0.879282000

1 1.916305000 2.712365000 2.197907000

1 1.836153000 1.903373000 0.633709000

1 5.114261000 0.902294000 2.811318000

1 5.928122000 2.486173000 2.792744000

1 1.665319000 -0.456696000 0.495612000

1 1.666705000 -2.699798000 1.526821000

1 2.078937000 -4.147855000 3.468512000

1 2.792853000 -4.434424000 5.822154000

1 3.472024000 -2.445230000 7.163571000

1 3.404044000 -0.196607000 6.181610000

1 5.393976000 4.127627000 4.400889000

1 4.064393000 4.930482000 6.326815000

1 2.113888000 4.712068000 7.801398000

1 0.033333000 3.539146000 8.455516000

1 -0.692805000 1.509320000 7.201643000

1 0.657424000 0.641376000 5.346259000

1 5.934048000 5.187412000 -3.585683000

1 4.688269000 0.290459000 0.319635000

1 3.123281000 4.179169000 -0.461239000

6 3.832277000 5.431591000 1.292715000

8 3.334832000 6.157362000 2.047844000

35 6.905407000 4.421383000 1.021347000

[Fe(PNP-*i*Pr**,**BIN)(H)(CO)Br] (**6B**).

26 4.109285000 4.338979000 -0.039925000

15 4.840101000 5.855667000 -1.520142000

15 3.803069000 2.518276000 1.183995000

7 4.917585000 2.998646000 -1.386069000

7 5.478119000 4.793694000 -2.733975000

7 4.395803000 1.306748000 0.100717000

6 5.430715000 3.427420000 -2.560586000

6 5.904584000 2.541286000 -3.539263000

6 5.843841000 1.178769000 -3.271815000

6 5.338134000 0.719936000 -2.061021000

6 4.884918000 1.669836000 -1.134935000

6 3.765895000 7.080225000 -2.472787000

6 3.108371000 6.466103000 -3.719621000

6 2.696807000 7.674434000 -1.534702000

6 6.354576000 6.862359000 -1.042975000

6 7.167428000 7.413683000 -2.223899000

6 6.001481000 7.970155000 -0.036480000

6 2.126522000 1.863994000 1.681295000

6 4.776993000 2.164859000 2.749856000

6 2.216955000 0.535968000 2.383943000

6 1.915979000 -0.649406000 1.658483000

6 2.054834000 -1.890570000 2.231681000

6 2.530954000 -2.023373000 3.561505000

6 2.718193000 -3.299428000 4.158956000

6 3.213550000 -3.416963000 5.436749000

6 3.549453000 -2.254172000 6.170903000

6 3.370727000 -1.002858000 5.624823000

6 2.844415000 -0.840290000 4.311160000

6 2.654206000 0.449214000 3.706902000

6 4.004450000 2.527180000 3.994648000

6 4.312550000 3.733101000 4.677879000

6 3.604932000 4.119869000 5.789391000

6 2.524679000 3.335330000 6.268324000

6 1.756832000 3.739152000 7.394156000

6 0.686705000 2.991001000 7.826448000

6 0.334945000 1.803922000 7.140399000

6 1.064035000 1.379827000 6.052082000

6 2.189702000 2.117989000 5.586515000

6 2.969901000 1.710416000 4.451479000

1 6.303480000 2.920768000 -4.473124000

1 6.199532000 0.467734000 -4.011754000

1 5.291789000 -0.337254000 -1.826251000

1 4.436931000 7.887809000 -2.793938000

1 3.841374000 6.128546000 -4.458839000

1 2.476906000 5.617565000 -3.444670000

1 2.480139000 7.223424000 -4.202790000

1 2.140305000 8.455846000 -2.064933000

1 1.993878000 6.896391000 -1.225691000

1 3.125324000 8.127284000 -0.635867000

1 6.961898000 6.112848000 -0.522033000

1 6.604540000 8.150200000 -2.807943000

1 8.065644000 7.918984000 -1.851163000

1 7.501811000 6.620380000 -2.899826000

1 6.919960000 8.423352000 0.353042000

1 5.418172000 8.771360000 -0.502864000

1 5.433156000 7.585194000 0.814325000

1 1.683122000 2.638910000 2.314078000

1 1.540275000 1.826889000 0.760918000

1 5.027436000 1.099173000 2.741117000

1 5.705821000 2.734476000 2.673704000

1 1.551348000 -0.556613000 0.638672000

1 1.806682000 -2.787304000 1.669482000

1 2.466864000 -4.184770000 3.580253000

1 3.355087000 -4.397541000 5.881751000

1 3.954788000 -2.350650000 7.174051000

1 3.636889000 -0.121633000 6.196848000

1 5.125508000 4.350793000 4.306782000

1 3.855078000 5.042641000 6.305990000

1 2.027909000 4.661063000 7.902324000

1 0.104306000 3.312579000 8.684938000

1 -0.522067000 1.225169000 7.472783000

1 0.775654000 0.473641000 5.531997000

1 5.830776000 5.122879000 -3.621309000

1 4.308551000 0.317601000 0.296733000

1 5.476283000 4.446964000 0.604561000

6 3.526483000 5.488058000 1.156721000

8 3.191106000 6.251001000 1.963165000

35 1.844344000 3.999030000 -1.174856000
